# Multi-Cloud Resource Management Techniques for Cyber-Physical Systems

**DOI:** 10.3390/s21248364

**Published:** 2021-12-15

**Authors:** Vlad Bucur, Liviu-Cristian Miclea

**Affiliations:** 1 MassMutual Romania, Record Park, Strada Onisifor Ghibu 20 A, 400185 Cluj-Napoca, Romania; vbucur1@gmail.com; 2 Department of Automation, Faculty of Automation and Computer Science, Technical University Cluj-Napoca, 28 Memorandumului Street, 400114 Cluj-Napoca, Romania

**Keywords:** cloud computing, AI, machine learning, neural networks, multi-cloud, cloud storage performance, self-driving cars, autonomous vehicles, resource management, API development, cyber-physical systems

## Abstract

Information technology is based on data management between various sources. Software projects, as varied as simple applications or as complex as self-driving cars, are heavily reliant on the amounts, and types, of data ingested by one or more interconnected systems. Data is not only consumed but is transformed or mutated which requires copious amounts of computing resources. One of the most exciting areas of cyber-physical systems, autonomous vehicles, makes heavy use of deep learning and AI to mimic the highly complex actions of a human driver. Attempting to map human behavior (a large and abstract concept) requires large amounts of data, used by AIs to increase their knowledge and better attempt to solve complex problems. This paper outlines a full-fledged solution for managing resources in a multi-cloud environment. The purpose of this API is to accommodate ever-increasing resource requirements by leveraging the multi-cloud and using commercially available tools to scale resources and make systems more resilient while remaining as cloud agnostic as possible. To that effect, the work herein will consist of an architectural breakdown of the resource management API, a low-level description of the implementation and an experiment aimed at proving the feasibility, and applicability of the systems described.

## 1. Introduction

Large scale machine learning and, more generally, AI applications need a large-scale approach to resource management. Resource management is understood to mean both hardware needs, especially storage and processing needs, in the case of machine learning, and software needs. In terms of software resources, robotics and AI applications need an easily scalable and maintainable framework that enables ease of use, distribution, instantiation, and can accommodate an ever-expanding set of business requirements [1].

Before presenting our approach to a multi-cloud resource management architecture it is important to understand the current state of the art in terms of resource management in the cloud. One of the most common uses of large-scale resource management systems in enterprise software today is for managing media files. Specifically, for self-driving cars, storing images to help with machine learning is of crucial importance. Media files have a particularity that other types of files do not in the sense that they are very large, they cannot necessarily always be compressed or split into smaller chunks, and they are a cornerstone of our daily lives [2]. Media files are an essential tool that many AIs, and implicitly robots, are trained on by using visual imagery [3]. The result of this cavalcade of information is that robots are learning to do things such as drive, in the case of self-driving cars, understand emotions, manage traffic, facial recognition, or even gardening [1]. An AI’s ability to perform beyond its normal constraints of decision trees and cascading logic rests on its ability to have quick access to as much information as possible. Hence why the need for multi-tiered cloud architecture for streaming video or images is already there as discussed by Eduardo Gama et al. in his article on fog/cloud architecture for streaming [4].

The article discusses a three-layered architecture, using a single cloud network that is obfuscated by a fog layer to keep the implementation hidden from the end process [4]. This setup addresses end customers and is subject to the laws and specifications of various European and international telecommunications standards [4] but is a good starting point in understanding how a resource management system can be used to handle large amounts of resources. Basically, the end user communicates via one of their Internet connected devices to hardware that is typically “user friendly”, such as 5G or telecom networks [4]. The packages then pass through a network of servers that forward the requests onto a database layer which finally retrieves the requested footage and streams it back to the user [4]. The proposed solution would then use services to connect all these layers together and ensure that multiple service implementations, perhaps one for each streaming interface, could work together [4].

Another approach to handling such large amounts of data or managing resources, such as instances of VMs, Lambda expressions or load balancing is to, in fact, let the AI work not only for the end user’s benefit but for its own benefit as well. In this situation, a possible implementation would be the use of intelligent agents to retrieve information, data mine or do language processing [5]. The agent’s behavior is motivated by programmed internal or external factors and the agent can take split second decisions as to the number of resources to divert or allocate from or to a process [5]. This is useful in and of itself in any hardware configuration, but it becomes especially useful in the context of cloud computing. Since cloud computing is distributed, parallel, cost sensitive, performance sensitive and nearly ubiquitous it forces developers to optimize the way they use resources.

A third proposed architecture, specifically for autonomous vehicles, consists of using a Hadoop cluster in the cloud to store very large amounts of data [6]. According to the authors, Google’s self-driving solution stores roughly one gigabyte of sensor data per second and this is obviously a big challenge for any software developer to handle [6]. The proposed solution would funnel all data sources through Flume, a tool used for collecting a large amount of streaming data to a Kafka cluster, which will then in turn send the data to Hadoop [6]. The HDFS (Hadoop Distributed File System) would then stream the data to another Kafka cluster which would be used by applications to consume the data [6]. A convolutional deep learning neural network is used by the vehicle to analyze the shifting in the pixels of the input image [6]. Using a combination of local respective fields, shared weights and biases and a pooling layer the car will detect a completed image [6]. The article further establishes a need to quickly process and store large amounts of data, usually as images, in a reliable fashion.

In *A Cloud-Connected Autonomous Driving System*, Wenfu Wang et al. highlights a three-part system for autonomous vehicle applications using a cloud layer, an application layer and a vehicle layer [7]. The article addresses several concerns over the architecture of such an application including safety related concerns regarding cloud availability, latency and security [7]. While cloud services are heavily used in autonomous driving applications, with a whopping 78% of participants in a survey answering positively when asked about a service-oriented architecture in a 2019 article [8], there are still many valid concerns about having a single point of failure. The solution proposed by Wang et al. is to harness the hot backup solution implemented by most cloud providers to ensure continuity of service for the application [7]. However, this solution does not protect in case of provider wide blackouts, nor does it consider the time required to run the backup routines and throttling procedures for distributing the data to the different servers provided by the cloud vendor. While it is true that most of these tasks are done in the background and the application is rarely affected by loss of data, it is nonetheless not a full proof solution.

Works presented in this article as well as many others that deal with cyber-physical systems show a desire to move to a cloud environment that is many times conditioned by complex security and availability needs. However, when leveraged together with complex machine learning mechanisms the multi-cloud is a boon to AI development [9]. The rest of this paper will explore ways in which a resilient and scalable multi-cloud architecture can be used to manage resources, costs and data integrity while enforcing safety requirements by providing more than a single point of failure.

This paper will explore ways in which resources can be managed and optimized in a multi-cloud environment using an architecture suited for large-scale, resource heavy operations undertaken by various applications and services. We hope to provide a blueprint for a possible solution on how to implement such a large-scale resource management system in a multi-cloud environment. To that end we will be focusing on identifying parallels between current single-cloud implementations and patterns that could be used in the multi-cloud along with a layered architecture, a realistic implementation and a mock proof-of-concept of a multi-cloud application. A series of challenges and possible issues will be presented before concluding.

## 2. Architecture

This section will provide an overview of the architectural choices adopted by the authors in implementing this resource management approach. It will contain definitions of ambiguous terms, an analysis of possible approaches and it will offer a high-level view on the flow of the entire resource management process.

### 2.1. Terminology

The definition of “resource” in computer programming is not ubiquitous nor is it entirely clear. In the *Apple Developer Guide* a resource is defined as “data files that accompany a program’s executable code” [10]. Indeed, this definition is one that is commonly used in colloquial programming jargon and equates “resources” to files holding data. However, a cloud platform does not just hold data, but it also provides system resources for manipulating and handling that data. The United States National Institute of Standards and Technology defines a computing resource as “an operating system abstraction that is visible at the application program interface, has a unique name, and is capable of being shared” [11]. It further goes on to clarify that, in the context of the document in which the definition appears, files, programs, databases and even external media are considered resources. Finally, in *The Kubernetes Resource Model (KRM)*, Brian Grant defines “resources” as Kubernetes pods, which are configured using YAML or JSON files [11]. A Kubernetes pod consists of an entire Docker container, therefore making the “resource” effectively unbounded, capable of holding both data and the applications that could manipulate it. Kubernetes does not limit itself to Docker containers though, but instead offers solutions even for non-containerized applications such as object storage in the cloud or serverless operations.

We, the authors, feel that given the lack of clarity on what a “resource” is understood to mean in computer programming, a clarification needs to be made on how that term will be used in the context of this article. Hereinafter, the authors understand “resource” to mean any hardware, software or data used in the process of running an application. The broadness of the definition stems from the complexity of cloud systems, where data is not just static, but can be dynamically modified by various calls to the cloud’s API. One example of this are AWS Lambdas, chunks of code used in Serverless computing that enable developers to run an application without provisioning for it or managing servers [12]. Lambdas can be triggered by calling any number of AWS services, from provisioning for new VMs to uploading files into S3 storage. Therefore, it stands to reason that an application which would make use of cloud storage, for example, would need to account for other types of resources used, outside of just the data storage requirements.

A “resource-management system”, therefore, needs to handle more than just balancing the loads of data upload, download or transformation from one server to another. It needs to account for the duration of time spent executing serverless or cloud-side operations. Additionally, it will need to consider if the data it manages the resources for is to be transformed upon upload or not, and if it is, what the acceptable timeframe for a response is. Based on this knowledge, we define a “resource-management system” as being an encompassing ecosystem that an application uses to carry out its business logic. It is understood that the rest of this article will accept the definitions of “resources” and “resource-management system” as defined by the authors.

### 2.2. Approach

There are two approaches to creating an API that manages multiple, simultaneous connections to various cloud providers: through a messaging service or as a dependency. The most common approach to working with distributed systems is Representational State Transfer, known as REST. The architectural style was first proposed by Roy Fielding and it presented a set of constraints. At the core of any RESTful service lies this set of constraints, which are treated more like suggestions and not enforcements as they would be in a SOAP architecture [13]. The degree to which those constraints are followed indicates the application’s Richardson Maturity Model [14], as defined in “*REST in Practice—Hypermedia and Systems Architecture*” and then popularized by Martin Fowler in a 2010 article [15]. Due to these very lax constraints only about 0.8% of web services are fully compliant with REST [16]. Even so, the basic constraints of statelessness, usage of HTTP action verbs and data transfer structure have become synonymous with distributed computing.

Using a REST based architecture greatly reduces the onboarding or learning process for a new API. HTTP calls using basic CRUD (Create, Read, Update, Delete) commands return a predictable pattern of results. No installation or declaration of dependency is required in any one project, any API using REST can be easily scaled, HTTP is language agnostic (as are JSON and XML) and tools for documenting an API’s functionality are abundant. However, REST does have some drawbacks, the most significant of which are its statelessness and the obfuscation of internals. 

Since REST is stateless, responses cannot contain resources. In the case of large media files this immediately becomes a problem because the file needs to be converted into a byte stream and then saved to file. This a costly operation, especially when resources need to be available synchronously. Furthermore, not all files are supported by MIME classification, which is widely used by REST. This leads to complications if the file is requested in a browser as the process that will implement the API will need to handle converting the file into something the end customer can see in their browser. 

**Figure 1:**
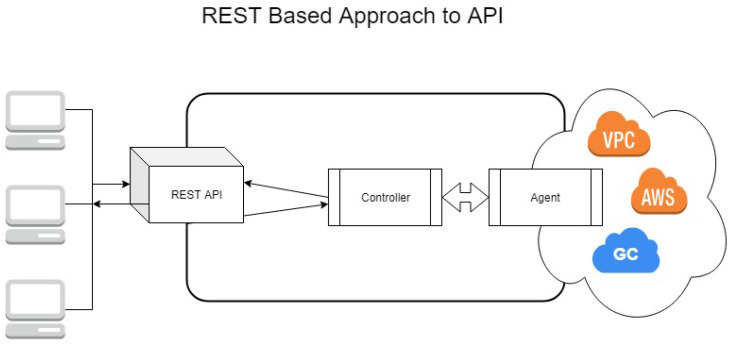
REST API Architecture with Multi-Cloud Application.

Finally, there is the issue of transparency. A REST API intentionally hides the implementation from the calling process (see Figure 1). This makes the debugging process considerably more complicated. In a case where a distributed storage strategy [16] would be employed to distribute file information over multiple clouds, debugging could become extremely complicated. Additionally, developers would be unaware during development and debugging of what is happening when they add a resource into the cloud through a REST call. This is in fact one of the biggest issues with using cloud services today, hence why solutions have been developed to debug locally. The most well-known of these is LocalStack, which mocks an AWS cluster on the developer’s local machine [17].

The alternative to using a REST based API would be to use a library as a dependency and then call the API methods provided by that library. There is a significant caveat to this approach, specifically that an API designed to be used as a dependency is not language agnostic and needs to be declared in every project that uses it. However, this approach allows for greater visibility and customization from a development standpoint, which is why the rest of this paper will focus specifically on a more traditional, stateful implementation of a multi-cloud API. 

**Figure 2:**
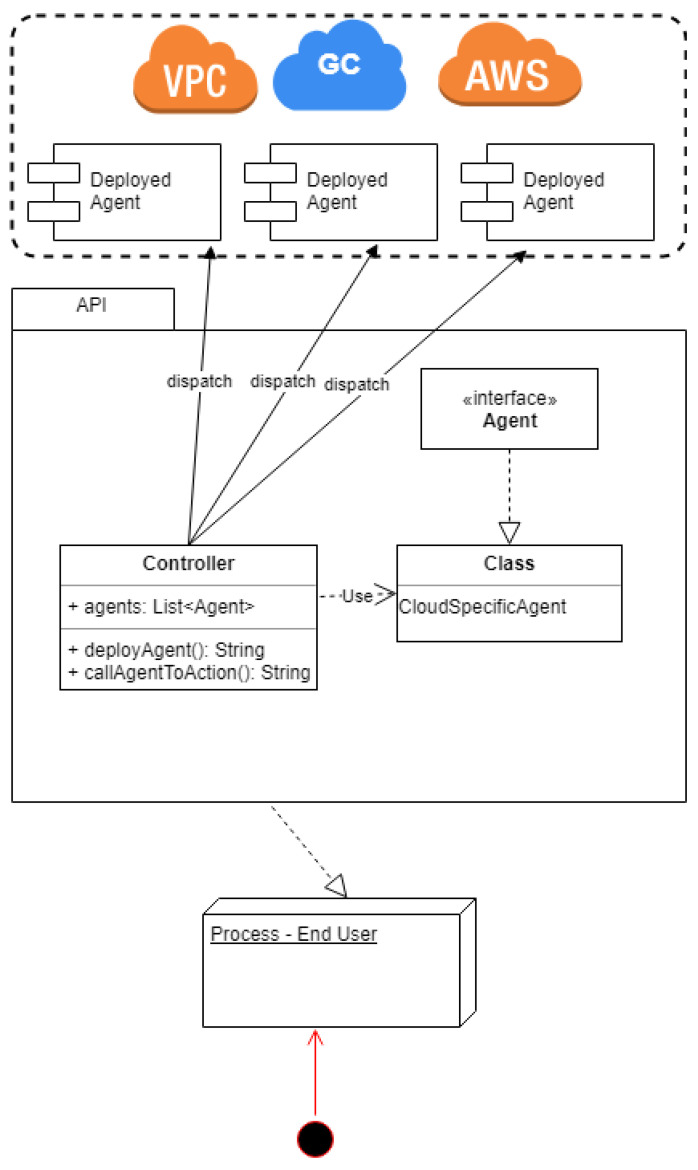
Resource management in the multi-cloud: simplified layered architecture.

Figure 2 shows a combination of a simplified layered architecture using agents—independently deployed applications that run on the cloud’s side—to monitor and communicate with each cloud. The API will need to provide compatibility with each of these providers separately and any others not listed. This is a common practice in many flexible ecosystems, even today, and it provides genericity and vendor agnosticism, while also allowing for customized plugins.

An example of such an architecture is Java’s JDBC API [18], an interface layer that is used to communicate with various types of relational databases. Each database provider implements the JDBC API interface and tailors the implementation to their specific relational database solution. The finalized product is delivered to customers as a plugin that is declared as a dependency in the project and is used for CRUD operations, transaction management and security profiling for that specific database. 

Kafka provides yet another example of this agent-based approach with its suite of Connectors: individual applications that handle one connectivity scenario. For example, the Kafka Connect FileStream Connectors are software build independently of the core Kafka cluster that allow developers to easily transfer data from Kafka to a file. Confluent, the parent company that operates the Kafka commercial license, offers dozens of these connectors that can be used for all types of read or write operations from or to different sources [19]. In addition to the commercially available connectors savvy developers can create their own connectors, either by using the code bases provided by Confluent through an open-source program, or by writing their own from scratch. This gives Kafka an incredible amount of flexibility and scalability.

### 2.3. Functionality

Adaptability to multiple cloud solutions is a baseline requirement for any application or API working in a multi-cloud environment. Providing an easily extendable solution then becomes key to helping expand the reach of the API itself. Making the API scalable and generic allows any new cloud provider to simply add a plugin to work with their version of the cloud. Additionally, the API is also exposed to enterprises that are using it, allowing them to create their own custom plugins or connectors. Finally, all these abstractions will make the cyber-physical systems using the API better managers of resources by making them easily scalable and not reliant on any one cloud provider. Rather, the systems will simply rely on the abstraction of a concept whose implementation is handled, behind the scenes, in the API layer.

The API will communicate with each separate cloud using agents, instead of plugins or just simple connectors that ferry data. These agents need to be lightweight, relatively autonomous and deployable on command should the need arise to redeploy them. 

By lightweight it is understood that agents should use as little of the managed resource as possible and that they should avoid complex implementations which would make them cumbersome to use. In essence they should do three things: return metrics from each cloud provider, query for a resource and carry back the results of requests made by the controller. Each agent will need to have a vendor specific implementation so that it can handle requests and responses successfully between the cloud provider and the controller.

The agent requires some degree of autonomy as it will need to be deployed on the fly as more resource requests are made to a different cloud provider. This is not at all dissimilar to how a cloud resource management system works, such as Kubernetes, where dockerized services and applications can be autoscaled and dynamic deployment is orchestrated based on the needs of a specific application. The difference between a Kubernetes implementation and the agents’ approach, however, is that the agents will be dynamic and not be deployed in a cluster, so that they can work on multiple clouds, on demand. To ensure seamless deployment a possible solution would be to dockerize the agent packages, thus ensuring that they are deployed in fully sustainable environments. Furthermore, dockerizing the agent deployment will make it possible to deploy agents programatically, without needing to previously set up each agent with a cloud provider.

Finally, the agents need to be redeployable easily. Albeit a simple requirement, the implementation behind the redeployment process could be quite complex. Agents need to realize that they are no longer connected to a counterparty, which is difficult to measure in certain situations. A scenario that would need to be accounted for in an automatic deployment environment would be the split-brain scenario.

**Figure 3:**
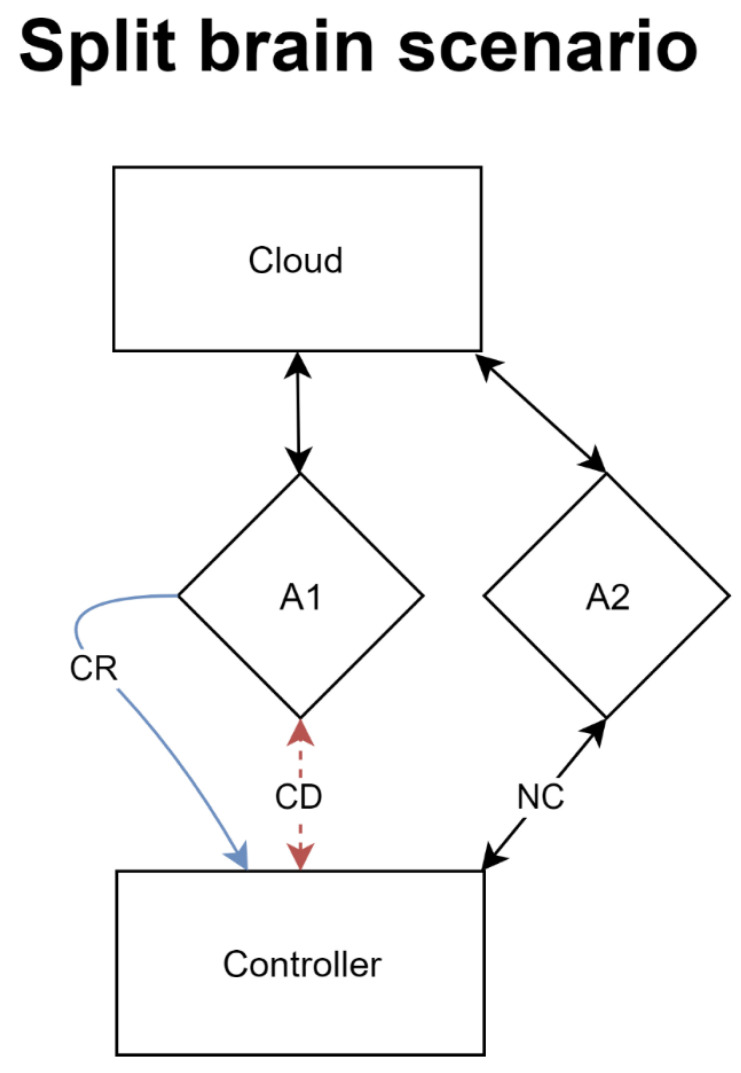
Split brain scenario where a connection is dropped from only one side.

In a split-brain scenario, exemplified in Figure 3, the agent loses the connection to the controller but not the cloud. The agent fails to report back to the controller, which then prompts a fail-safe trigger to deploy a new agent and attach it to that cloud provider. However, once the first agent’s internet connection comes back online it too will attempt to connect to the controller. Such a situation must be carefully managed by both the controller and the agent to ensure that partial information is not sent back to the controller nor that the controller is flooded with events and messages from duplicate services.

The controller is a component of the API and acts as the brains of the application, much like it does in a model-view-controller architecture. It is the element that coordinates the deployment of agents, or their redeployment, as needed. The controller also interprets the data it receives from the agents as well as the data it receives from the process, via the API wrapper, and it makes the decisions as to which resources to contract, when and for how long. Ideally the controller would also benefit from a predictive resource-requirements model that will make it easier for it to automatically anticipate a resource intensive task, based on the type, and frequency, of calls it receives from the API. For the controller to benefit from a predictive model it will need to be supplied with regular performance benchmarks from the clouds and cloud services the application is using. This is an optional parameter which is setup during customer implementations as the benchmarking is done automatically and can hinder performance somewhat, or interfere with high throughput tasks.

Both the controller and the agents are part of the same package. The controller is cloud agnostic while the agents are adapted to a cloud solution. The agents do not have direct visibility of the controller. They act only as a bridge between the controller and the cloud so that the former has select visibility of the cloud platform’s performance metrics and is able to issue commands to it (and get back responses). Since this approach is not REST based, the controller will not be language agnostic. The API, in its entirety, will be declared as a dependency by the project and the confirmation that a resource was acquired or a request was posted to the server will be an internal communication.

On its end, the entirety of the API communicates with both the process and the controller, albeit in different ways. The process does not necessarily need to directly communicate with the API: it will declare it as a dependency and then use the provided methods to make requests. If a request fails, the API can return an exception or an error. The API does not necessarily need to acknowledge every request, especially since all the communication is done inside the process and is, most likely, asynchronous. Assume a resource intensive operation, such as a self-driving car going from point A to point B, is being processed. The process makes requests to the API for resources as needed, it remains blissfully unaware of the API’s implementation, it only needs the resources. Furthermore, the process anticipates a heavy resource load coming up and it preemptively requests the API to provision for that heavier load, for example when the car encounters traffic on the way to its destination. The API then receives these requests from an application that implements it, and it sends them to the controller requesting more resources. Based on the requests made by the process, provisioning for a heavier incoming load, the controller allocates resources accordingly. Both the API and the controller need to work asynchronously, because multiple threads will carry out the request and send answers back to the API at various times during the execution of said request.

The last element of Figure 2 is the process. The process is not part of the architecture and does not need to know any implementation details about any of the levels above it because of two main reasons. Firstly, as is the case with all encapsulation, the fewer elements that have access to an implementation the easier it is to isolate and track any potential bugs. Secondly, the API needs to be generic in order to accommodate multiple agent types. 

In essence the architecture presented in this section is not entirely different from any type of large enterprise-level architecture, but it attempts to make use of intelligent resource rationing and modern micro-services design patterns to ensure that communication is seamless at all levels of the hierarchy. That is, of course, if said levels even require communication. One of the key goals in having a resource management system is that not all levels need to communicate to reduce the need to separately configure load-balancing solutions. What makes this approach different from a traditional load balancer though is the fact that it grants higher availability, the performance management is not solely relegated to a certain provider or on premises servers and it heavily decreases vendor reliance.

## 3. Implementation

In this part of the article, we will present the real-world implementation of the API as well as an experiment using mocked stubs of code to highlight how that implementation would work in a production environment.

### 3.1. Development

Regardless of the implementation details the primary goal of any good API implementation is that it needs to be as generic as possible. In REST, making the API scalable and easy to use means making it language agnostic.

There are many cases of this type of implementation, most notably REST over HTTP which is used for a variety of web service interactions and requests [20]. When implementing an API this way the user only needs to make calls to the API itself, usually in plain text, and they will receive the information they requested without the need to memorize many methods. When using REST, it is as easy as calling a PUT command for the API to know that an application has signaled an intent to send something, somewhere. Similarly, the API implementation in this case will just require the process to ask for more resources and then the backend processes will file the request with the controller. Even in the case of a dependency-based architecture, the preferred approach when designing an API is to use logically named methods, generally relying on HTTP action verbs, be as stateless as possible and be easily extendable.

Any application also needs to be scalable, able to either handle multiple threads from a predetermined thread pool, with extra resources on reserve, or to be idempotent to be easily deployable in multiple instances. Additionally, the API will need to be easily maintainable to give it the required flexibility to add support for even more cloud services in the future. At the beginning it is expected that the application will only have support for a limited number of cloud service providers but in the future this number might grow. Currently many cloud providers offer a myriad of compilers, virtual machines and programming languages to use when doing cloud development. However, not all cloud providers are nearly as generous, and some have a limited number of programming languages, compilers, or tools at a developer’s disposal. Furthermore, not all the libraries available for every language are quite the same, so when the level of abstraction increases those differences in implementation become very much minor. This issue will need to be handled programmatically and is independent of the architecture chosen. One possible approach to fixing it would be to closely follow the SOLID programming principles, specifically Interface Segregation [21]. 

Interface Segregation is the *I* in the SOLID principles and it states that the absolute minimum amount of code should go into an interface [21]. If a class needs a wide range of functionality, it would just implement multiple interfaces, as multiple interface inheritance is allowed by most object-oriented programming languages. The agent would, using this approach, need to implement only the interfaces it needs and provide only the functionality that a specific cloud provider is offering. Another approach would be for the controller to act as a very generic wrapper on top of the agent, exposing only the most basic operations that are provided by all cloud providers. Specific operations would be handled by an extension of the controller that is injected with an agent as a dependency. Through casting or some other means, such as reflections, the cloud specific controller uses the agent dependency to call those methods specific to the agent and, implicitly, the cloud provider.

At the lowest level, however, the implementation will be very similar to most load balancers currently available. The application will measure the throughput from a virtual machine, or a service, deployed in the cloud and report the performance data. The controller will interpret the data and compare it to previously established benchmarks, it will check the current availability of the platform and resource requirements. The controller will then make a call as to whether to scale on a different cloud provider or simply scale with the current one. Should the application decide to stick with the current provider, but should more resources still be needed from said provider, the controller will delegate load balancing duties down the line to a dedicated cloud management platform like Kubernetes. This is because Kubernetes will use specific end points created for each cloud provider to use those cloud provider’s controllers to measure ingress and then decide on how many new instances to request [22].

**Figure 4:**
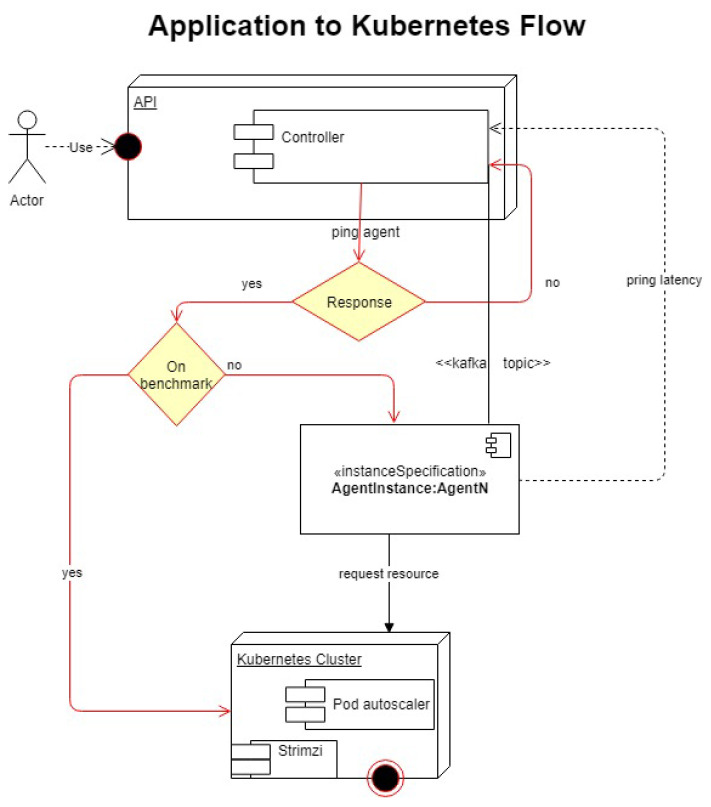
Diagram depicting a complete, high-level, resource request workflow using the API.

Figure 4 shows a high-level summary of the scenarios in which the application will decide whether to request resources from the same provider or whether to switch to a new provider. The diagram assumes that the controller was preloaded with information about baseline performance data in the form of benchmarks. 

In Figure 4, the actor, for example a machine learning application that uses AWS SageMaker to deploy and manage to process data through Apache Spark, calls an operation from the API to augment resources. The allocation process is done, programmatically, through the controller keeping in mind the data request made by the Actor. At this point, the controller then establishes which vendor to query for more resources, if AWS is unresponsive or below benchmarks. The controller needs to be aware that the new vendor it will provision from is above the current performance threshold. The controller will only be able to establish which vendor is quickest by being aware of each vendor’s performance benchmarks for various operations.

Initially the controller will receive a request from the API for a new resource. It will then ping one of the agents, the priority of which can be selected through a customer-level implementation. All requests made by the controller to the agent(s) will be stored in Kafka topics to ensure traceability and data integrity. After it pings the agent and establishes that the agent is alive and can respond, the agent will then attempt to get a response from the cloud provider. Once it gets a response it will forward the response to the controller, which will then proceed with the next step in measuring whether the response is within benchmark parameters or not. If it is within the parameters, the controller will tell the agent to call on Kubernetes to begin the process of allocating new resources. Should the response not be within the limits set by the benchmark, or a custom threshold, the controller will then call on a new agent. The order in which each provider’s agent will be called will be established by priority settings.

Should benchmarks not exist, the controller will ignore this step and default directly to Kubernetes and have it request more resources. Finally, in a situation where the agent does not respond at all, the controller will then call on a new agent. This call will loop several times or for several seconds, both of which can be customized from the configuration file, until it decides that, for reasons external to the runtime, it can no longer communicate with the agents.

It is important to note that all the settings that a user might require to configure their implementation of the API need to be editable by the developer. As a result, the application steers away from hardcoded values for all its components, including the benchmarks. Users and those who implement the API in their process’ logic will be allowed to configure the number of times the controller pings the agents, either by time or by number of tries, whether the benchmarks are used or not, what benchmarks are used, if they are loaded up from a static database or whether they are generated on the fly and more. Ideally, setting these options should be achievable through multiple means, just like they are for any modern application. Settings could be consumed from Kafka topics, static files (.ini, .yml, .xml, .json, etc.) or in a purely programmatic way, created and populated at compile time.

### 3.2. Data Integrity

Ensuring data keeps its integrity is a complicated process for even the most basic load balancer or queuing solution, even those that are running on a basic client-server architecture and are not using distributed computing. More importantly, in the case of autonomous vehicles data integrity can be, quite literally, a matter of life and death. Therefore, it is very important to address this topic of utmost importance in its own separate section. 

Before delving deeper into the finer details on how the API will keep data structured as intended it must be mentioned that the application presented in this article alone will not be able to, and should not, handle data integrity by itself. It is preferred to use the integrity guarantees provided by a cloud provider’s services individually. This is ideal especially since the agent only communicates with the Kubernetes cluster, which then brings up the requested cloud service using preconfigured pods. Moreover, the separation of concerns is crucial to developing reliable scalable software and it empowers an application to use the best available technology for any given task. No one software developer or corporation can be highly skilled at each of the many complex tasks an application undertakes. Using specialized tools for each task is, therefore, a forgone conclusion.

Keeping all of this in mind the best solution to ensure data integrity, in this approach, would be a Kafka cluster specifically configured to run once the API is deployed in production. Out of all the Kafka implementations available on the market the ideal, lightweight, minimally configured solution would be Strimzi. Strimzi is a Kubernetes native Kafka cluster that can be deployed in minutes, is highly configurable, has built in security and uses dedicated nodes to deploy the cluster on [23].

Having a Kafka cluster that is so easily deployed means that the resource-management API can leverage the incredibly robust Kafka ecosystem to ensure data integrity. Since the cluster is so malleable and easily configurable the API will only need to create topics for storing information that is sensitive to data corruption or disruption. The controller will create Kafka topics on startup for all the data deemed sensitive and all agents will consume these topics and then make requests to the Kubernetes cluster. Since the Kafka cluster offers multiple backups of its brokers, topics and partitions, the application that works with it needs to only consume or produce to a fixed endpoint. Thusly, if one agent has lost its connection to the cloud, the application will continue to place records in that topic, and once a new agent comes online that agent will then consume the data from the topic and forward the request to Kubernetes. Once the request has reached Kubernetes, it will then provision a resource and the application’s workflow will continue uninterrupted.

Since Kafka topics are by default just byte arrays, they can be configured to accept all types of data. For example, topics that will be used by the application to request resources could just use plain strings to make the request, and once an agent is deployed those requests are forwarded to the Kubernetes cluster which will allocate more resources to the requesting application.

Agents will use the provided Kafka offsets to know where to start picking up messages from the topic. If the message is not consumed by an agent, the offset will not be committed and therefore the next agent will pick up from where the last one left. Should the record be consumed by an agent, but a resource would not be provisioned the agent will place the negative response from Kubernetes in a dead letter queue (DLQ), natively provided by Kafka, and the controller will then pick up the record from that DLQ and rehydrate the original topic. This ensures that the next agent that starts consuming form the topic will also fulfill the failed resource provisioning attempt in addition to carrying out any further requests.

### 3.3. Experiment Setup

To better illustrate the functionality of the API described herein an experiment has been engineered that makes use of a multi-cloud resource management system to send data to three cloud providers.

The three different cloud providers chosen for this experiment are: Amazon Web Services (AWS), Google Cloud (GC) and LocalStack. While AWS and GC are well-known commercial cloud providers that need no introduction, LocalStack might be a bit more obscure. LocalStack is a Dockerized container that simulates a cloud environment on a local machine. The cloud implementation simulated by LocalStack is AWS along with 24 of its services. Using a LocalStack instance as part of the experiment is meant to simulate private, on premise, clouds, which are a common practice in much of software development, including applications for self-driving cars. Additionally, LocalStack provided a way to run test scenarios related to interruptions of service, which are infrequent in production-level cloud environments and hard to simulate on demand.

The test environment was set up to use IAM based authentication for AWS and GC. The keys and two separate profiles, one for each cloud provider, were setup on the local test machine. Operations were performed through the SDKs provided by Amazon and Google separately using the stored credentials. The third profile, the local profile provided by LocalStack, had no security constraints.

LocalStack was deployed through Docker Desktop on a Windows 10 based machine using WSL 2 shell for running Linux natively on Windows. The distribution of Linux used to run the containers was Ubuntu 20.04. Containers were allocated four CPUs and ten gigabytes of RAM as well as sixty gigabytes of storage space. The configuration of the computer running the LocalStack container was as follows:

- Intel Core i7-7700K @ 4.20 ghz (8 cpus)
- 16 GB of DDR4 RAM @ 1.20 ghz
- Samsung SSD 860 EVO M.2 2TB
- Windows 10 Pro 10.0.19042 build 19042

The cloud service under test was each of the cloud providers’ proprietary cloud storage solution. In the case of AWS, S3 was used, while in case of GC the experiment was conducted using Cloud Storage. The choice of which cloud service to use came down to ensuring a fair and level playing field during the experiment. The application was also designed to test a commonly used resource in development, cloud storage. Furthermore, running the tests on cloud storage abstracted the provisioning of resources as both S3 and Cloud Storage are hardware agnostic. 

Both types of storage options provided the ability to run tests in the same region, namely the Eastern Coast of the United States (us-east-1). Finally, cloud storage is ubiquitous in cloud developed. It is used intensively, is highly distributed and very flexible which makes it a great choice for storing data to be used in machine learning, as is the case of self-driving cars. 

To run the experiment a simple application was developed, using each cloud provider’s SDK, to run a PUT operation into a predefined bucket. In S3 a specifically designed PutObjectRequest class was used for both AWS and on LocalStack, since this was the only class that would return a REST-like response. The Google Cloud SDK provided a create() method, part of a generic Storage class, that was implicitly built like a REST API. The method used PUT operations and returned a response on either success or failure. 

The application was deployed from the main class, in as lightweight a manner as possible, and used these methods to send batches of 100 files to each bucket, without any delay, to simulate a real-life workflow where speed is of the essence. After recording the baseline performance of each cloud, the application was modified to throw random exceptions every three operations to gauge the speed at which the backup cloud would pick up the failed PUT request. Response times were measured on successfully completing the PUT operation, exception and backup operation completion. Java was used throughout the coding of the application and Instant.now() was the measurement of choice for calculating the timing of each operation. Instant.now() returns the GMT to the nanosecond. This was useful given the time zone differences between the local machine and the cloud hosted storage.

### 3.4. Experiment Results

The results of the experiment are presented, in milliseconds, in Table 1. Baseline values represent the total duration of each upload operation when done using solely one cloud. For each of the multi-cloud solutions the first provider is the one that “fails” while the second one is the backup. All times in the table represent the start time after the first successful PUT call and the end time after the confirmation that the operation was successful.

From Table 1 it is immediately clear that there are major differences between the speed of upload among cloud providers. LocalStack is the quickest one to complete the upload, which is entirely due to it being locally hosted and not requiring an internet connection. The second quickest, by quite a large margin, is AWS S3 which took less than half the time to upload 100 files as it took GC Cloud Storage. Notably, however, all cloud providers, in both multi-cloud and single cloud, had a 100% success rate in uploading the files with no data losses. It is important to keep this in mind when analyzing the multi-cloud performance.

Turning to the multi-cloud measurements one can observe clear slowdowns when using multiple providers. Most notably, when using a combination of LocalStack as a primary cloud and AWS as the backup the time required to upload the data jumps by approximatively 434%. This appears to be an outlier, however, since the implicit delay of sending information over the network, with authentication, is considerably larger in the baseline results as well.

Analyzing the AWS to GC and GC to AWS figures though shows some interesting facts. When using a combination of AWS S3 as primary cloud and GC as backup the slowdown in GC is significant enough to increase the duration of upload from the AWS baseline by more than 52%. However, when reversing the primary and backup clouds the difference between the GC baseline and the combined multi-cloud approach between GC and AWS shows only a 15% increase in upload time. This shows that, in a highly optimized production environment, some cloud provider’s services are considerably better than others in some key areas. Google Cloud Storage is significantly slower than AWS S3 and even in a basic experiment where a record is sent to the S3 bucket roughly 33 times out of 100, the delay, even with authentication and internet connection, is small enough to encourage developers to use both clouds at the same time and optimize the usage of each storage solution.

Another important element of the experiment was represented by the interruptions in service tests, which showed similar results to the values in Table 1. The interruptions in service could only be tested using LocalStack but it’s important to note that, despite a 5000-ms delay (a result of automatic retries in the AWS SDK), the PUT operations were successfully completed on LocalStack and no data was lost because of the service outage.

Most importantly though, the results show that there is a clear use case for an API to manage resources in the multi-cloud, since there are considerable differences between each cloud offering even in the most basic of services offered. The API implementation outlined in this section would make use of benchmarks, such as the baseline figures in Table 1, to determine which cloud offering is the best suited for managing a certain resource, be it storage, serverless operations or provisioning. Furthermore, the experiment proves that a workable model of the API could be used successfully in a production environment because of its resilience and ability to quickly switch between cloud providers, seamlessly, granted that runtime environments are properly configured.

Last, but not least, it compels the authors to discuss the ever-important issue of pricing. While prices for these PUT operations were relatively low there were still significant differences in costs between the two commercial cloud providers. In AWS customers have access to a limited number of free operations. What constitutes as “free” is determined by AWS based on data usage, uptime, compute power or time windows. Google Cloud has a different pricing strategy. It offers prospects $300 worth of “free credits” [24] but those credits are only applicable to some products [25] and are time limited, lasting only 90 days [26]. Crucially though, while both providers opt for a “pay as you go” pricing, AWS offers completely free data transfers into S3 and only charges for anything over 1 GB/month for transferring data out of S3 [27]. This represents a significant cost-optimization opportunity for using a multi-cloud environment to manage financial resources used by an application. Software using low amounts of storage or that does not need to download data from a bucket could use S3 indefinitely, for free. In the case of Google Cloud Storage, the same software would be charged for every operation that goes beyond the $300 ceiling provided by the trial.

## 4. Challenges and Future Improvements

Most of the challenges related to this application and its architecture are caused by the complexity of working with distributed computing. A few of these challenges will be presented in the current section.

The first and arguably least damaging issue is that of API maintenance. Any API, whether language-agnostic or as a dependency needs to be thoroughly documented, using all the techniques listed here:

- Extensive in-code documentation, usually in the form of comments or other, language specific, notations (i.e., JavaDocs).
- Instruction manuals for developers that need to implement the API.
- API documentation, available online, fully featuring in-code examples, in multiple languages, if required.

Generally, the problem with this approach is that semantics, descriptions and purposes change throughout a product’s lifespan and it is quite possible that different naming conventions, semantic complexity or inconsistent documentation will lead to longer review processes, slower commit rates and more convoluted concepts inside the API itself. The task of documenting an API, providing examples, and keeping them updated is a major overhead for any organization. Examples must be clear, concise and act as a catch-all for at least the most basic of operations.

The second challenge that will be faced by any implementing application is the issue of individual, provider-level, SLAs. Harmonizing SLAs and implicitly costs, in the form of both actual costs and perceived costs, such as losses with downtime or server outages, becomes very complicated when the implementation and responses are hidden.

Usually developers, businesses and processes know of a failure as soon as it happens because something stops working. In the case of the application discussed here, when something stops working the controller simply goes to the next cloud provider in the list. When using this application for demonstrative purposes and simply using the lowest tier of hardware, usually free, this is not much of an issue because switching from one free VM to another involves almost no costs. Though, this is not quite the case. 

The problem is that VM configurations among cloud providers are not identical and in a situation where a provider is switched during a resource intensive machine learning process, for example, the “same” hardware might be more expensive when using a different provider. That is because even if the switching is kept to the same tier of machine the actual hardware offered by different cloud providers differs significantly. The solution here would be to configure which tier machine is to be used for each operation, and for each vendor, if a need ever arises to switch vendors.

Another considerable hurdle are the benchmarks since without updated benchmarks the application becomes a simple load balancing mechanism. Instead of balancing resources between different VMs on the same cloud though it distributes resources to various VMs of different cloud providers.

Benchmarks need to be kept up to date and need to be done regularly to ensure that when the controller decides on whether to switch from one cloud provider to another, based on a response time comparison between that cloud provider and its benchmark, it does not do so using old information. Although there’s myriad ways to solve this issue, dropping the benchmarks all together and working solely on pings to and from a cloud provider might invalidate the resource optimization process. Another solution would be to employ micro-benchmarks [28] which would cut down on the overhead and would be able to provide quickly available snapshots of a vendor’s performance profile.

Based on just the ping alone the controller cannot make but a split-second decision on which server has the better internet connection, but not whether that server offers better object storage speeds, more CPU cycles or higher uptime. These decisions need to be made based on a comparison between the benchmark numbers, crosschecked with the expected response time, to ensure that resources are used more efficiently when switching to another vendor.

Before concluding the Challenges section several observations about the proof-of-concept experiment must also be raised. One of the most difficult things to test in the current cloud environment are service outages. Ensuring continuity is at the base of this multi-cloud approach to managing resources and must, as a result, be tested. Currently most cloud providers offer incredible uptime guarantees making it hard to test with service outages in a production environment. These tests are not just important because they prove resilience, recoverability and guaranteed uptime but because they can be an important window into studying data integrity and distribution in multi-cloud systems. While testing locally with a mock cloud was insightful in demonstrating that the API can easily recover from a catastrophic failure it was not enough to prove what would happen in a real-world scenario where an application consumes data from a bucket that suddenly goes dark. The data that was stored in that bucket might immediately become obsolete and the consumer application will need to work only with the backup bucket from that point onward. To demonstrate what impact this would have on an actual application the experiment will need to be expanded upon to use the full resource management ecosystem, including Kafka for backup and data transfer, Kubernetes for provisioning, an application that uploads significant data and another application that works with that data in a semi-real-time fashion.

Moreover, the experiment only deals with one type of cloud resource: cloud storage. A resource management system should not be understood to mean a data transfer utility, but rather an ecosystem for managing the multitude of cloud services on offer. An enterprise level proof-of-concept would, as a result, need to manage more than just storage resources and would need to verify that the integrity of any operation made by the application is kept throughout. 

The final limitation of the experiment is that testing was only run with two cloud services at once. While that goes to prove the concept of managing resource in a multi-cloud environment, in the future the experiment should be expanded upon to include three or more cloud providers. Additionally, custom agent implementations should also be considered for testing. This would expand the coverage of testing to encompass data integrity and the recovery processes of applications that use these resources.

## 5. Conclusions

When it comes to resource intensive operations AI, robotics and machine learning are the most likely candidates to use the multi-cloud. Cyber-physical systems are poised to become forerunners in terms of hardware usage in the following years. While the concept of the multi-cloud is novel, the huge amount of data already in circulation and the petabytes of data created every minute need to be efficiently sifted through, processed, organized, and then fed to AIs for them to grow. Additionally, all these systems, be they self-driving cars, shop attendants, gardening robots or even intelligent devices, will need to become increasingly more aware of their surroundings and act on split second decisions.

Developers currently need to provision ahead of time for resources they anticipate will be used. They need to manually call, in some way or another, be it programmatically or through over provisioning, the resources they need. This is costly, error prone and raises multiple safety concerns.

The application presented in this paper hopes to ease the developers’ jobs when it comes to resource allocation and stability. Such an application will also help researchers by making it possible to harmonize costs and increase the capacity of AI development and research in the future. The end goal is to empower cyber-physical systems in a way that makes them part of our daily lives and helps us solve complex issues, and not just in their current state, as limited, experimental applications with considerable drawbacks.

## Figures and Tables

**Table 1:** Speed of upload using single and multi-cloud.

Provider	Start	End	Start Δ End
Baseline LocalStack	2021-07-24T20:45:00.4838	2021-07-24T20:45:02.5394	2055.6
Baseline AWS	2021-07-24T20:44:21.9280	2021-07-24T20:44:36.0373	14,109.3
Baseline GCloud	2021-07-24T20:21:22.4182	2021-07-24T20:21:52.4176	29,999.4
AWS w/LocalStack	2021-07-24T17:19:27.8162	2021-07-24T17:19:43.9570	16,140.8
LocalStack w/AWS	2021-07-24T17:14:20.9198	2021-07-24T17:14:30.0327	9112.9
AWS w/GC	2021-07-24T20:43:03.7763	2021-07-24T20:43:30.8339	27,057.6
GC w/AWS	2021-07-24T20:41:30.3472	2021-07-24T20:42:05.3864	35,039.2

## Data Availability

Not applicable.

## References

[1] Ilager S., Muralidhar R., Buyya R. Artificial Intelligence (AI)-Centric Management of Resources in Modern Distributed Computing Systems. Proceedings of the 2020 IEEE Cloud Summit.

[2] Aazam M., Huh E.N. Inter-cloud Media Storage and Media Cloud Architecture for Inter-cloud Communication. Proceedings of the 2014 IEEE 7th International Conference on Cloud Computing.

[3] Li Z.N., Kuang P., Zhang T., Yan H.R., Gu X.F. Deep Reinforcement Learning Based Game Decision Algorithm for Digital Media Education. Proceedings of the 2019 16th International Computer Conference on Wavelet Active Media Technology and Information Processing.

[4] Gama E.S., Immich R., Bittencourt L.F. Towards a Multi-Tier Fog/Cloud Architecture for Video Streaming. Proceedings of the 2018 IEEE/ACM International Conference on Utility and Cloud Computing Companion (UCC Companion).

[5] Qiu L., Li K. The Research of Intelligent Agent System Architecture Based on Cloud Computing. Proceedings of the 2016 12th International Conference on Computational Intelligence and Security (CIS).

[6] Kumar S., Goel E. Changing the world of Autonomous Vehicles using Cloud and Big Data. Proceedings of the 2018 Second International Conference on Inventive Communication and Computational Technologies (ICICCT).

[7] Wang W., Deng H., Sun M., Pan Z. A Cloud-Connected Autonomous Driving System. Proceedings of the 2020 IEEE 5th International Conference on Cloud Computing and Big Data Analytics (ICCCBDA).

[8] Banijamali A., Heisig P., Kristan J., Kuvaja P., Oivo M. Software Architecture Design of Cloud Platforms in Automotive Domain: An Online Survey. Proceedings of the 2019 IEEE 12th Conference on Service-Oriented Computing and Applications (SOCA).

[9] Salman T., Bhamare D., Erbad A., Jain R., Samaka M. Machine Learning for Anomaly Detection and Categorization in Multi-Cloud Environments. Proceedings of the 2017 IEEE 4th International Conference on Cyber Security and Cloud Computing (CSCloud).

[10] Apple Inc. (2016). Resource Programming Guide. https://developer.apple.com/library/archive/documentation/Cocoa/Conceptual/LoadingResources/Introduction/Introduction.html#:~:text=and%20Localization%20Guide-,About%20Resources,and%20into%20more%20appropriate%20tools.

[11] U.S. Department of Commerce Technology Administration–National Institute of Standards and Technology (1993). Minimum System Requirements for Multi-User Operating Systems. https://csrc.nist.gov/glossary/term/resource.

[12] Amazon Web Services (2021). AWS Lambda. https://aws.amazon.com/lambda/.

[13] (2004). World Wide Web Consortium (W3C). https://www.w3.org/TR/soap/.

[14] Webber J., Parastatidis S., Robinson I.S. (2010). REST in Practice-Hypermedia and Systems Architecture.

[15] Fowler M. (2010). Richardson Maturity Model. martinfowler.com. https://martinfowler.com/articles/richardsonMaturityModel.html.

[16] Neumann A., Laranjeiro N., Bernardino J. (2018). An Analysis of Public REST Web Service APIs. IEEE Trans. Serv. Comput..

[17] LocalStack (2021). What Is LocalStack?. https://localstack.cloud/docs/getting-started/overview/.

[18] Zhang Y., Zhang L. JDBC-based middleware applications in instant message systems. Proceedings of the 2014 2nd International Conference on Systems and Informatics (ICSAI 2014).

[19] Confluent (2021). Connectors to Kafka. https://docs.confluent.io/home/connect/overview.html.

[20] Roger C.L. (2020). Building Web Services the REST Way. http://www.xfront.com/REST-Web-Services.html.

[21] Martin R.C. (2000). Design Principles and Design Patterns.

[22] (2020). Kubernetes. https://github.com/kubernetes-sigs.

[23] Strimzi (2021). Strimzi Overview Guide. https://strimzi.io/docs/operators/latest/overview.html.

[24] Google Cloud (2021). Google Cloud Pricing. https://cloud.google.com/pricing.

[25] Google Cloud (2021). Solve Real Business Challenges on Google Cloud. https://cloud.google.com/free.

[26] Google Cloud (2021). Google Cloud Free Program. https://cloud.google.com/free/docs/gcp-free-tier/#free-trial.

[27] (2021). Amazon Web Services, Amazon S3 pricing. https://aws.amazon.com/s3/pricing/.

[28] Scheuner J., Leitner P. Estimating Cloud Application Performance Based on Micro-Benchmark Profiling. Proceedings of the 2018 IEEE 11th International Conference on Cloud Computing (CLOUD).

